# The Development of Sex Reassignment Surgery in Thailand: A Social Perspective

**DOI:** 10.1155/2014/182981

**Published:** 2014-03-19

**Authors:** Prayuth Chokrungvaranont, Gennaro Selvaggi, Sirachai Jindarak, Apichai Angspatt, Pornthep Pungrasmi, Poonpismai Suwajo, Preecha Tiewtranon

**Affiliations:** ^1^Division of Plastic & Reconstructive Surgery, Department of Surgery, Faculty of Medicine, Chulalongkorn University, Bangkok 10330, Thailand; ^2^Department of Plastic Surgery, Institute of Clinical Sciences, Sahlgrenska Academy, Sahlgrenska University Hospital, University of Gothenburg Gröna Stråket 8, 41345 Gothenburg, Sweden; ^3^Preecha Aesthetic Institute (PAI), Bangkok 10110, Thailand

## Abstract

This paper reviews the development of gender reassignment in Thailand during the period of 1975–2012, in terms of social attitude, epidemiology, surgical patients' profile, law and regulation, religion, and patients' path from psychiatric assessment to surgery. Thailand healthcare for transsexual patients is described. Figures related to the number of sex reassignment surgeries performed in Thailand over the past 30 years are reported. Transsexual individuals are only apparently integrated within the Thail society: the law system of Thailand in fact, does not guarantee to transsexuals the same rights as in other Western countries; the governmental healthcare does not offer free treatments for transsexual patients. In favor of the transsexual healthcare, instead, the Medical Council of Thailand recently published a policy entitled “Criteria for the treatment of sex change, Census 2009.” The goal of this policy was to improve the care of transsexual patients in Thailand, by implementing the Standards of Care of the World Professional Association of Transgender Health. Currently, in Thailand, there are 6 major private groups performing sex reassignment surgery, and mostly performing surgery to patients coming from abroad. Particularly, the largest of these (Preecha's group) has performed nearly 3000 vaginoplasties for male-to-female transsexuals in the last 30 years.

## 1. Introduction

A comparative study of international centers performing GRS has been previously published [1]. This survey reported on the standards and policies used in 1995 by 19 clinics located in Europe and North America. Today, surgical treatment (as well as the entire transsexual healthcare) is standardized in University Hospitals and is based on the Standards of Care (SOC) published by the World Professional Association for Transgender Health (WPATH) [2–6]. The overall goal of the SOC is to provide clinical guidance for health professionals to assist transsexual, transgender, and gender nonconforming people with safe and effective pathways to achieve lasting personal comfort and to maximize their overall healthcare. This assistance may include primary care, gynecological and urological care, reproductive options, voice and communication therapy, mental health services, and hormonal and surgical therapy.

Usually, the patient is referred from the General Practitioner (GP) to a mental health professional with a special interest in GD and eventually to a physician who can prescribe hormonal therapy. After a minimum of 12 months of continuous experience of living in an identity-congruent gender role under the control of the mental health professional and a minimum of 12 months of hormonal therapy, with a confirmed diagnosis of GD, the transsexual patient is referred for genital surgery [2]. At the end of the gender reassignment, patients still need to be provided with health assistance for life, to monitor the general health conditions and the surgical outcomes.

Dr. Preecha Tiewtranon and Dr. Prakob Thongpeaw performed the first male-to-female sex reassignment surgery in Thailand at the Chulalongkorn University Hospital (CUH) in Bangkok in 1975 [7]. At that time, the society had negative thinking about the transsexual patients and the surgical procedure [7].

Previously, no paper has reported on the past and present social and health care provided to transsexual patients in Thailand. The scope of this paper is to review the development of sex reassignment in Thailand during the period of 1975–2012 in terms of epidemiology, religion and social attitude, law and regulations, surgical patients' profile, and patients' path from psychiatric assessment to surgery. We were not able to find any other studies describing the development of sex reassignment surgery in other cultures or countries.

## 2. Epidemiology

In Europe, the most recent epidemiological studies and review about transsexualism report prevalence ranging from 1 : 11,900 to 1 : 45,000 for male-to-female persons (MTF) and 1 : 30,400 to 1 : 200,000 for female-to-male (FTM) persons [8], with an increasing number of patients seeking assistance in the recent years [9]. Particularly, in The Netherlands the prevalence of people wishing to receive hormonal or surgical therapy because of the incongruent gender identity is 0.6% for biological males and 0.2% for biological females [10].

In Asia, a recent report calculated the rates in Japan to be 1 : 25,000 for male-to-female and 1 : 12,000 for female-to-male [11].

Another study from the University of Singapore showed that the transsexuals number in this island is 35.2 per 100,000 inhabitants (1 : 2,900) for FTM and 12 per 100,000 (1 : 8300) for FTM [12].

Finally, the unpublished studies from Winter [13, 14] are calculating a prevalence of 0.3 to 0.6% MTF persons in Thailand.

Currently, the Chulalongkorn University, Bangkok, Thailand, is in the process of granting a study and collecting data on the prevalence of transsexualism in Thailand. Since it is possible to observe and find transsexual people in any public place such as school, universities, and any working place, a much higher incidence of transsexualism in Thailand is expected, compared to the data reported in the world literature.

## 3. Influence of Religion and Social Attitude on Gender Reassignment

Thailand is roughly 95% Buddhist. Winter and Udomsak have already published an overview on the position of Thailand transsexual within the Buddhist religion and society [15].

As in other South-East Asian societies, nonnormative gender categories form part of the indigenous cultural tradition [16], with the common belief until the beginning of last century of the existence of three sexes: male, female, and male-female [17].

Jackson (1988) [18] reports that the Buddhist Vinaya text (a code of conduct for monks) identified four main sex/gender categories: males, females,* ubhatobyanjanaka* (hermaphrodites), and* pandaka* (males displaying a variety of other nonnormative anatomies or sexual preference) [15].

Today, the terms transgender and transsexual are seldom used in Thailand. Instead,* Kathoey*, a word originally used todenote hermaphrodites is used today to describe a male-to-female transsexual [18].

Unlike Christians, Buddhists cannot point to specific religious laws or teachings forbidding homosexuality, transsexuals, or gay marriage. One of the fundamental teachings of Buddhism is tolerance of those who act differently or hold different views. At first blush, this tolerance (if not acceptance) would seem to extend to transsexuals in Thailand. Transsexuals, in fact, are integrated into everyday life, and physical or verbal assault on transsexuals in public is extremely rare [17].

Again, Buddhism may play a role. While the Buddhist focus on tolerance does in part shape Thailand society's tolerant view of transsexuals, the Buddhist principle of karma may provide an alternative explanation: Thai people believe that people are born* kathoey* because they are being punished on this life of their misdemeanor of a previous one [17, 19].

The standard karmic tale, in fact, is that transsexuals were formerly “playboys” in their former lives and, as a result of breaking so many lovelorn hearts of women, they were imposed the ultimate punishment, making them a woman trapped in a man's body, forever doomed to unrequited love. Therefore, they are a group to be pitied, not protected.

In the past, in fact, the Thai society had negative thinking about the transsexual individuals and the surgical procedure; particularly, the Thai society disliked patients' appearance and their overacting manners; furthermore, transsexual people were considered to have low education and no taste and were believed to work mainly in the sex industry [20].

Thai people also believed that the result of the surgery was not good, giving lots of complications [20].

It was not long ago (December 1996) [15] when a transsexual student enrolled in an education program at a public university murdered a female friend; in response to this, another well-known higher education institution in charge of Thailand's teaching universities banned transgender and homosexual students from attending their teacher training facilities.

At the time, transsexuals were variously referred to as “sexually deviant” and “sick…mentally” by members of the institute, and they were considered of having a bad influence on children. This argument was simply keeping in line with the Department of Mental Health, which at the time considered homosexuality a mental disorder. Subsequent pressure from both Thai and Western gay rights groups soon forced a repeal of the ban.

Up to day,* kathoey* have become so prominent a part of modern Thai culture that the authorities have taken steps to reduce their profile, for example, making it more difficult for them to work as teachers or tour guides and advising television stations to curb MTF appearance on shows [15].

According to a study by Dr. Winter, Hong Kong University students showed a marked male antipathy towards male gender variance [21].

At the opposite, more recently, several transsexual individuals became successful in their careers, especially in the entertainment and mass media industry such as the actors, singers, reporters, and even models.

Transsexual beauty pageants, such as Miss Tiffany's Universe and Miss Alcazar, are held in Thailand and televised nationally each year.

Today, Thailand appears to live up to its worldwide reputation as a place where transsexuals can experience greater freedom and acceptance than in other nations.

Particularly, transsexuals' appearance and manners seem to the Thai population much more similar to the way ladies are acting and behaving. At the same time, the sex surgical conversion got more and more the reputation of being successful, both domestically and internationally. In fact, parallel to the explosion of the tourism market (90's) in Thailand, more foreigners were coming to Thailand to receive sex change operations. The Thai population has been witnessing this medical tourism for the past 20 years [20].

In spite of this new Thai face, a closer look at its society and culture, however, still reveals a society with a decidedly mixed view of transsexuals [22].

## 4. Law and Regulations

### 4.1. Law and Society

Thailand legal system reflects the Thai society [23].

As far as the government of Thailand is concerned, male-to-female transsexuals are legally men. Transsexuals cannot legally change their gender on their ID cards, leading to problems with potential employers. Many employers do not want possible complications involved with hiring a transsexual if an equally qualified “normal” person can be hired. Because of this, the vast majority is relegated to occupations traditionally held by women, that is, waitresses, hairdressers, makeup artists, and vendors, even if they are university graduates. Transsexuals' birth gender must remain the same on their passports as well; a fact that can lead to confusion and unwanted scrutiny at border crossings and immigration checkpoints. Thailand also prohibits same sex marriage, meaning that when the partner of a transsexual dies, the deceased's family receives any or all assets [24].

In the past years, there have been some positive developments since the transsexuals and the gay community in general have achieved some legal gains. In 2002, in fact, the Department of Mental Health, under intense pressure from the gay community, removed homosexuality from its list of mental disorders. This decision helped to pave the way for the Thai military to announce in 2005 that it would discontinue its practice of dismissing transsexual and gay recruits for having “a severe mental disorder” and the subsequent announcement in March 2008 that the military would be adding a “third category” for transsexuals [24]. This third category would allow transsexuals to be dismissed from service due to “an illness that cannot be cured in thirty days”, thereby removing the scarlet letter of “mental disorder” from their service records-records that must be provided at each job interview and with each loan application.

### 4.2. Regulations for the Treatment of Gender Reassignment

Until 2009, there was no definite rule to regulate gender reassignment in Thailand; nevertheless, some plastic surgeons (as the authors of the present manuscript) were still following the SOC as suggested by the WPATH, as for the criteria to select and treat transsexual patients. However, until 2008, there were many cases of castrations to teenagers, performed by nonurologists and nonplastic surgeons, and this made the social and the mass media upset and panic [23, 24].

As a consequence, pressure was put on the Thailand Medical Council to regulate the medical practice for the treatment of transsexual patients; subsequently, the Thailand Medical Council published a policy entitled “Criteria for the treatment of sex change, Census 2009”, which was effective from November 25, 2009. Among the criteria, patients have to be over 20 years of age, or they can be between 18 and 20 and have consent of at least 1 guardian. Also, patients should have documents to approve their surgery from 2 different psychiatrists. For other details, the SOC of the HBIGDA/WPATH should be followed, including furthermore, the real life experience for at least 1 year [23, 24].

## 5. Thailand Health Care for Transsexual Patients

In spite of the fact that the Thailand Medical Council posed regulations on the treatments of transsexual patients, Thailand Government hospitals generally do not offer free treatments (psychological assessment, hormonal or surgery) to transsexual patients.

For this reason, Thaitranssexuals seek for all their gender treatments privately.

Most of the Thai transsexual patients do not visit the psychiatrist at the onset of the gender dysphoria: in fact, they do not believe that psychiatrists can be of any help to them, but only for signing for the diagnosis, which is allowing the surgeon to proceed with the surgery. Further to this, the quality of the care organization offered at the psychiatrist services (and other medical services) in the Government hospitals is presenting with long waiting queues; finally, very few psychiatrists in Thailand are currently interested in this field, and these psychiatrists are practicing in private settings. As a consequence, most of the transsexual patients visit the psychiatrist only when they decide to get the surgery done.

The hormonal treatment is a very weak point within the treatment, too: very few endocrinologists in Thailand have experience in this field, and patients prefer to listen to senior fellows of their society, accepting suggestions passed to each other as word of mouth, rather than giving their trust into endocrinologists. As a consequence, nearly all the transsexuals use hormones, but very few of them are under the care of endocrinologists. Hormones are usually bought directly from the pharmacy; no prescription is required.

Thai transsexuals mostly start with the hormonal treatment when in secondary school (14-15 years old). The popular hormones used are the contraceptive pill such as Progynova; Diane-35; Premarin; once a day; and/or Progynon/Proluton injection every 1-2 weeks. Some patients also add Androcur.

Sex reassignment surgery is usually performed when patients are in their 20's, and at this age most of the patients have received hormonal therapy for at least 5 years.

Nearly all the surgical treatments are performed privately. The Government finances surgical treatments rarely, and for teaching purposes only. These surgical treatments are exclusively performed at CUH and, more recently, at Lerdsin General Hospital in Bangkok. Even in these cases, surgery is not offered free of charge, but patients have to pay for some of the hospital costs. In 2012, 8 vaginoplasty operations in MTF transsexuals were performed at CUH (see Table 1). Three to four mastectomies in FTM transsexuals and 2 penile reconstructions (usually metaidoioplasty, with less than 10 procedures performed in the last 20 years) are also performed at CUH per year.

For transsexual patients coming from abroad and seeking private sex reassignment surgery in Thailand, letters confirming diagnosis of gender dysphoria from psychologists/psychiatrists from the patient's homeland are regularly accepted.

Currently, while in the University Hospitals all patients are from Thailand, within the private sector the ratio of foreigner to Thailand patients is 10 : 1 (see below).

Opposite to surgery for MTF transsexuals, surgery for FTM transsexuals is not common in Thailand even within the private sector.

### 5.1. Followups

Thai transsexuals receiving vaginoplasty are usually followed up until 1 year postop, while foreigner transsexuals are usually receiving their last followup with their surgeon 2 weeks postop; then, they are instructed to continue their care with endocrinologists or gynecologists in their home countries. They might come to Thailand for later followups if needed, but this is uncommon.

## 6. Surgeons' Profile

To date, there are about 20 Thai surgeons able to perform SRS. However, the highest number of the procedures (MTF only) in concentrated among 6 major groups are as follows: PAI (Preecha's Aesthetic Institute) (Bangkok), Suporn (Pattaya, Cholburee), Chettawut (Bangkok), Kamol (Bangkok), Sanguan (Phuket), and Yanhee (Bangkok).

By directly interviewing the surgeons involved in GRS in Thailand, we calculated the ratio between Thai and Foreigner patients surgeries operated in Thailand in the past years. All the above-mentioned Bangkok-based centers are included in this survey, except Yanhee.

Between 1985–1990, only 5% of the transsexual patients operated in Thailand were foreigners; at the opposite, 90% of the transsexual patients operated in Thailand in the period 2010–2012 were foreigners (see Table 2).

Now a day, there are at least 2 or 3 foreigner patients operated per day in Thailand, and receiving MTF-SRS.

Since 1985, the mean age of the Thailand transsexual patients is 27 years old (17–50).

Mean age of the foreigner transsexual patients is 46 years old (16–72).

Foreign patients resulted to have higher degrees (e.g. medical doctors, engineers, lawyers, police officers, etc.), more successful and stable in their profession, rather than Thai patients.

## 7. Conclusion

Since the first Thailand's sex change surgery in 1975, the Thai social attitude, law system, and regulations to treat transsexual patients have very much improved. Particularly, the WPATH SOC are full in place within the regulations presented by the Thailand Medical Council. In spite of these improvements, Thai transsexual individuals are not fully integrated within the society, the law system does not guarantee to transsexuals the same rights as in other Western countries, and health treatments for gender dysphoria (psychiatric assessment, hormonal and surgical treatments) are not provided free of charge within the umbrella of the Government Hospitals, but these are paid by the patients themselves. On the other hand, we believe that the way Thai transsexuals appear in the media and to the Western world, the current Thailand economy allowing for surgery at a cost cheaper than in Western country, and the experience progressively achieved by Thai surgeons over the past 30 years contributed to an increasing number of foreign patients coming and receiving gender reassignment surgery in Thailand.

## Figures and Tables

**Table 1:** Number of vaginoplasty procedures performed at Chulalongkorn University Hospital.

Year	Number of vaginoplasty procedures performed
1973–2007	Data not available
2008	12
2009	17
2010	9
2011	13
2012	8

Total	59

**Table 2:** Ratio of the Thai/Foreign MTF transsexual patients operated in Thailand.

Periods	Thai/foreigners
1985–1990	95%/5%
1991–1995	90%/10%
1996–2000	75%/25%
2001–2005	50%/50%
2006–2010	30%/70%
2006–2010	30%/70%
2010–2012	10%/90%
